# How Were European GPs/FPs Involved in the COVID-19 Vaccination Campaign? A European Questionnaire Study about the Experiences of the Vaccinations in 2021

**DOI:** 10.3390/healthcare12171785

**Published:** 2024-09-06

**Authors:** Imre Rurik, Péter Torzsa

**Affiliations:** Department of Family Medicine, Faculty of Medicine, Semmelweis University, Üllői út 25., 1091 Budapest, Hungary; torzsa.peter@semmelweis.hu

**Keywords:** COVID pandemic, Europe, family medicine, primary care, vaccination

## Abstract

Background: The SARS-CoV-2 pandemic has become the greatest public health challenge worldwide. Soon after the appearance of the virus in 2019, intensive efforts to develop vaccines were initiated, and by late 2020, delivery of vaccines for the targeted population as a campaign had started. Aim: Collect information from European Union countries regarding how and to what extent were family physicians (FPs)/general practitioners (GPs) involved in the vaccination campaigns in 2021 and how these were organized at the national level. Method: A short questionnaire was distributed through the secretariats of WONCA (World Organization of Family Doctors) Europe and the European Forum for Primary Care (EFPC). Results: In most of the countries, participation of FPs/GPs was compulsory. The vaccination was usually centrally organized by governmental authorities. In the beginning, registration (web-based) of patients was required, mainly at the national level. By the middle of 2021, vaccination on a walk-in basis became available in almost every country for the first immunization as well as for the booster injections. The remunerations of GPs/FPs differed; in some countries, no extra payments were offered. The Pfizer vaccine was used in all countries, while in nine countries, non-European Medicines Agency (EMA)-approved vaccines were also given in primary care settings and at vaccination centers. In some countries, professional homepages helped the GPs. The involvement of primary health care (PHC) providers did not correlate to the vaccination coverage of the entire population of the respective countries. It was the highest in the more developed countries with higher living standards, where participation of GPs was voluntary and appropriate financial incentives were offered to them. Conclusions: The vaccination campaign was a professional and logistic challenge and an excellent performance of PCH providers. Experiences gained could be used in the future to manage similar pandemic challenges.

## 1. Introduction

The pandemic of 2020, caused by the severe acute respiratory syndrome coronavirus-2 (SARS-CoV-2), was a major public health challenge worldwide. Practically none of the healthcare systems were prepared to manage the pandemic, treating thousands of infected persons and facing high mortality rates even in the most developed countries. After the outbreak, different restrictions were introduced in most of the countries based on physical distance-keeping, obligatory use of face masks, also frequently combined with curfews. It was clear to the scientific community as well as to governments that appropriate vaccinations would be the best way to resolve the pandemic. Pharmaceutical companies started intensive research to develop vaccines alongside enormous expansion of manufacturing capacities. Vaccines are a core strategy to reduce the transmission of SARS-CoV-2 and the severity of disease resulting from SARS-CoV-2 infection [1]. Several vaccines have been available to the public since late 2020 [2]. However, their success at the population level depends largely on whether countries can quickly achieve high vaccination coverage rates. Equitable distribution of and easy access to vaccines both among and within countries are crucial to avoid severe COVID-19 and to slow the development of potentially dangerous variants, for “no one is safe until everyone is safe” [3].

Since late 2020, vaccination campaigns started in many developed countries (North America and the European Union) after obtaining permission from professional agencies or governmental authorities. The following vaccines were authorized for use in the European Union: *Comirnaty* (BNT162b2, manufactured by BioNTech and Pfizer), *Nuvaxovid* (XBB.1.5 by the Novavax), *Spikevax* (mRNA-1273, by Moderna), and *Vaxzevria* (AZD1222, by AstraZeneca) *COVID-19 Vaccine Janssen* by Janssen. Others are still currently under rolling review: *Sputnik V* (Gam-COVID-Vac, Gamaleya Institute), *Vero Cell* COVID-19 Vaccine (HB02, Inactivated, Sinopharm), *Vidprevtyn* (Sanofi Pasteur), and *VLA2001* (Valneva). Except for the Janssen vaccine, all required a booster vaccination (3–5 weeks) after the first shot [4]. For the majority of the European population, vaccinations started at the beginning of 2021, and in the second half of the year, the third (booster) vaccines were offered by the health authorities.

Primary care providers (PCPs), family physicians (FPs), and general practitioners (GPs) are working in the closest settings to the population; they have the most information on the health needs and socio-economic conditions of their patients. Although the structure of primary care is different by country, they can play a vital role in improving access to vaccines against SARS-CoV-2 and their equitable distribution within individual countries [5]. Indeed, in most jurisdictions, GPs provide lower-barrier care than that offered by specialists and have a closer relationship with their patients than do many other providers. Thus, they can build trust in the vaccine and help overcome vaccine hesitancy [6]. Several countries have explored options to mobilize PCPs for this purpose. Germany, for example, included PCPs in its vaccination campaign in April 2021 and managed to double its daily vaccination rate within a day [7]. That same month, the US Department of Health and Human Services and the Centers for Disease Control and Prevention recommended that the jurisdictions should increase the proportion of vaccines allocated to primary care providers, with at least 60% of that proportion going to providers in socially vulnerable communities [8]. Most of the European countries had a priority list for their citizens, preferably for the older populations and for patients with multi-morbid conditions or malignancies.

Upon surveying the international professional platforms and actual correspondences of FPs/GPs (including social media), it becomes clear that primary care providers in European countries were involved differently in the vaccination campaign. We did not find relevant papers about it even in the most prestigious databases. The campaign itself was not among the topics of selected primary care publications [9]. European primary care organizations seem to be an appropriate source of information. WONCA Europe (W-E) has 51 national member societies from 41 countries [10], and the European Forum for Primary Care (EFPC) has many institutional and NGO members from 25 countries of the continent [11].

### Aim

We aimed to provide an overview of how primary care was involved in the COVID-19 vaccination campaign carried out in Europe, focusing on 2021 as the first year of vaccination. Our basic hypothesis was that FPs/GPs were involved in the vaccination everywhere, but differences could be between countries. While no similar study from other continents was found, we tried to focus on Europe only.

## 2. Method

Inclusion criteria: at least one well-informed key person of primary care from all countries of Europe (not only EU-member states).

Surveying is one of the most frequently used research methods in primary care. It works if the number of questions is limited and does not require much time to be completed; therefore, a short and easy-to-manage questionnaire (see Appendix A) was compiled by the authors and distributed through the secretariats of WONCA Europe (W-E) and the European Forum for Primary Care (EFPC) in January 2022. They provided it to the leaders (chairperson or secretary) of the affiliated PC organization. Ninety-five persons were targeted, fewer in countries being less active within these organizations. Responders were encouraged to disseminate the questionnaire among their colleagues. The questions focused on the involvement of GPs in their respective national campaigns, on the main executors of vaccination, the venues of provision, the required registrations of clients, the remunerations of GPs, the type of vaccines they used, and the presence of national web-pages focusing to the vaccination in PC settings.

Statistics: Distribution of answer options and data provided by the questioned professionals were presented only, without any specific statistical probe.

## 3. Results

A total of 51 questionnaires were returned, and 47 were appropriate for analysis. A total of 12 questionnaires were returned from 10 non-EU countries (Albania, Georgia, Israel, Kirgiz Republic, Norway, Serbia, Switzerland, Tajikistan, the UK, and 3 from Turkey). The number of EU responders (out of 28) was 21, often more than 1: Austria (4), Belgium (2), Bulgaria, Croatia, Czech Republic, Denmark, Finland, France, Germany (7), Greece, Hungary, Italy, Malta, The Netherlands (2), Poland, Portugal, Romania, Slovakia, Slovenia (3), Spain, and Sweden (2). Answers are presented mainly according to the questionnaire, sometimes restructured to fit better to the tables.

### 3.1. Participation

Participation of GPs was compulsory in Croatia, Czech Republic, Georgia, Greece (for the public salaried GPs only), Hungary, Serbia, Slovenia, Tajikistan, Turkey, and the UK. In addition to vaccinations, a prioritization list of high-risk patients was expected in The Netherlands and Norway. In Kirgizstan, public communication of the rationale for vaccination was also the duty of the GP. Some level of activity in public communication and patient recruitment was required in almost every country.

GPs participated voluntarily in Switzerland, Sweden, and Italy with differences between regions (counties/cantons). Voluntary participation—often with high rates of uptake—was characteristic in Austria, Denmark, Bulgaria, Belgium, Czech Republic, Finland France, Germany, Malta, Poland, Romania, and Slovakia. In Israel, GPs have to inform and mobilize their patients toward vaccination centers. Albanian and Portuguese GPs were expected to contribute to the vaccination points, established only for this purpose.

Injections were administered by nurses in Finland, Portugal, Spain, and Sweden, while in other countries both doctors and nurses were authorized to give the shots. Nurses performing vaccination were supervised usually by their own GP.

### 3.2. Main Executors

In most countries, the location for vaccinations was mainly the vaccination points (hubs, lines, busses), dedicated only to this purpose. Drive-through centers were operated in Romania. The main executors were the doctors (contracted or ordered to work here), often the FPs themselves. In some countries, hospitals served also as vaccination points (Austria, Greece, Hungary). Otherwise, GPs performed the vaccination mostly at their offices. In Israel, the Health Maintenance Organisations organized and executed the vaccinations with their staff.

### 3.3. Level of Organization

All of the countries paid priority attention to the campaign; therefore, it has been centralized by the governments. In some countries, regional coordination is operated mainly where federative state systems exist. (Bundesländern/cantons: Austria, Germany, Switzerland), regions: Belgium, Finland, Poland, Romania, Sweden, and Spain. In Poland and Romania, wider autonomy of regions and a high level of local/city autonomy were reported. The logistics of vaccines also differed. There were governmental deliveries or GPs had to order vaccines through the local pharmacy.

### 3.4. Registration’s Requirement

At the time when the vaccination of the population started, registration of patients at the national (web-based) level was required in Albania, Belgium, Bulgaria, Czech Republic, Denmark, Greece, Italy, Kirgizstan, Poland, Serbia, Slovakia, Slovenia, Tajikistan, Turkey, and Hungary, where the name, address, social security number, phone number, and email address of patients were collected by the government. Hungarian GPs had an enormous task to allocate their patients to hospital-based vaccination points. In all countries, the priority lists set up by the GPs considered the age of the patients, co-morbidities, and other risk factors.

In many countries (Austria, Belgium, Finland, France, Germany), patients had to register only locally at the nearby vaccination points/hubs/busses that were advertised for them. GPs were expected to facilitate their client’s registration. Later on, by the middle of 2021, vaccination on a walk-in basis became available in almost every country for the first immunization as well as for the subsequent booster injections.

### 3.5. Involvement in Respective National Coverage

In most of the countries surveyed, GPs and their nurses carried out the vaccinations, and they estimated their achievements in the vaccination of the entire population within a wide range. The self-reporting of the involvement of PHC providers in the vaccination was the highest in Serbia (close to 100%), Slovenia (95%), and Greece (80%). Between 40 and 60% was estimated in Austria, Bulgaria, Georgia, Germany, Hungary, Italy, Kirgizstan, and Turkey. Around 20–25% was calculated in the Czech Republic, Slovakia, and Romania, where the injections administered by GPs at the vaccination points were estimated as 50%. In The Netherlands and in Sweden, 5–10% was calculated, while practically none (1%) in Belgium and Israel. Wide differences between cantons were reported in Switzerland (5–30%).

### 3.6. Reimbursement

The remunerations of GPs/FPs differed. No extra payment was reported in Albania, Belgium, Finland, Israel, Kirgizstan, Portugal, Spain, Sweden, and Tajikistan. In Malta, only the private GPs received financing. GPs in the UK received their regular reimbursement for vaccination. The self-reported vaccination fees are listed in Table 1.

### 3.7. Vaccines Used

In most of the EU countries, only EMA-approved vaccines were used in primary care settings and at the vaccination centers. There were 9 countries where vaccines not approved by EMA were also used. The preferences for vaccines changed during the campaign in 2021. Table 2 gives an overview of the vaccine consumptions. Only the Pfizer vaccine was used in all countries.

### 3.8. Available Professional Websites

In many countries, official websites were established or previously existing platforms were updated with information for PHC providers. This was not the case in every country, and GPs were not always notified of them. Table 3 presents these homepages in the format provided by the responders and in alphabetic order of the countries.

## 4. Discussion and Conclusions

The vaccination campaigns were organized and managed not the same way in the European countries. There were differences between governmental organizations, registrations of patients, and obligations and remunerations of providers.

The COVID-19 outbreak has significantly changed all aspects of general practice in Europe. There were many changes in hospitals to isolate and care for a huge number of infected persons as well. There were changes also in medical education; moreover, the pandemic challenged doctors’ safety and well-being [12,13].

European primary care workforces were deployed differently in the vaccination campaign. In most countries, the majority of injections were administered by PHC providers, while in other countries, governments found different solutions. The preferred vaccines often changed by country during the campaign. After some unwanted side effects of the Astra-Zeneca vaccine were reported, its usage decreased in many countries [14]. There was also evidence of political influence over which vaccines were to be used. Countries that are closely linked economically or politically to Russia and China used vaccines originating from these countries in spite of the fact that these vaccines lacked approval by the EMA [15]. The different levels of involvement of PHC providers in the vaccination campaign were decided by health policymakers, and were based on the capacities of the respective health care system. The same considerations apply to the assignment of vaccination points. The establishment of a registration system helped to plan the vaccination, although in Hungary many personal data were collected by the government.

There were differences in the vaccination protocols and organizations within countries having federal state systems (Austria, Germany, Switzerland). This involved autonomy as well as responsibility at more local levels.

Some European countries have found solutions to increase their workforce in primary care. Other healthcare professionals were incorporated to support family doctors assuming their tasks, under their supervision and coordination, often in the vaccination centers. The European Commission played a crucial role in the purchase and distribution of the vaccines. The engagement of primary care in the vaccination campaign has had unequal participation in different countries, although the greatest burden has been managed by the government public health departments [16].

Public communication about the need for vaccination was an important duty of PHC providers. An additional task was the compilation of priority lists on the basis of individual risk factors for patients. At a later time, pressure diminished as vaccination became available on a “walk in basis”.

Involvement of PC and delegation of new tasks requires more payment [17]. Not all of the government or insurance companies offered extra remuneration for this during the campaign; thus, the incomes of the PHC providers differed between European countries [18].

The involvement of PHC providers did not correlate with the vaccination coverage of the entire population of the respective countries. In those where participation of GPs was mandatory, these figures were the following: Serbia (47%), Tajikistan (51%), Turkey (60%), Croatia (67%), Slovenia (68%), and Hungary (73%) [19,20,21]. The countries that could achieve the highest population coverage were those where the participation of GPs was voluntary and appropriate financial incentives were offered.

It seems that countries having better economic situations (higher GDP and living standard) could achieve higher population coverage during the campaign (Finland 90%, France and Denmark 92%, Norway 93%). It was not general, although it was almost 100% in Portugal and only 69% in Switzerland [19,21].

The vaccination campaign was a professional and logistic challenge for PHC providers in all countries. It was very effective in Europe [22]. The lessons learned will inform governments and public health authorities about what are likely to become regular national COVID-19 vaccination campaigns in the future. How to reach priority populations, how to track vaccination status, and how to ensure coverage at regional and national levels are all key questions that will inform us about the nature of public health and primary care/family medicine cooperation [23]. Convincing the target populations to manage their hesitations was an important issue as well [24]. The lessons learned in 2021 would be useful for the future as well. This infection will not disappear in the upcoming years and could return after genetic modifications [25]. COVID-19 infections already received less attention in 2022, although some Eastern and African countries had to face it. In 2023, the WHO chief declared an end to COVID-19 as a global health emergency [26].

Critical evaluation of public health policies is needed in many countries. High-income nations, national governments, donor agencies, and other relevant stakeholders must support the World Health Organization’s COVAX initiative to ensure fair, rapid, and equitable distribution of the vaccines to countries, irrespective of income level. Low- and middle-income nations must significantly invest in research, healthcare, vaccines, and drug development and must remain proactive in preparing against future pandemics [27,28]. We really hope that official bodies of all countries with low vaccination coverage are preparing for the future.

However, there was another problem that arose. Because of the COVID-19 issues, patients with other morbidities were neglected by the providers and often by themselves as well. We all have to fill this gap quickly, supported by the other players in the healthcare system.

## 5. Limitations

Unfortunately, not all the EU-member states were involved. We did not receive feedback from the Baltic countries and Cyprus. The ratio of vaccinated persons in PC settings is based mainly only estimation. We did not have access to official/governmental data and, therefore, had to trust the answers. Other difficulties were to understand national homepages in the local language. Only whole national coverage was presented on reliable websites. Different answers from countries with federal structures were not inconsistent; they reflected alteration between regions.

The protocols changed in many countries when the 3rd and 4th shots were introduced, often with different types of vaccine. Some of the questions could have a different meaning in the countries involved. Although we are grateful for the contribution of colleagues, some questions remained unanswered. Our aim was only to provide an overview for colleagues who are interested in this topic. We tried to share the experiences of the first year of nationwide vaccinations, and we did not want to compare national “*White books*” or national public health policy papers that may provide more in-depth information. We did not find a similar PC study in the literature; therefore, we were unable to compare PC experiences from other continents.

## Figures and Tables

**Table 1:** Remuneration of GPs for vaccination at their office or working at vaccination centers.

Country	for Each Injection [€]	Working at Vaccination Centers [€] Per Hours
Austria	20–25	150–160
Bulgaria	5	
Croatia	1.30	
Czech	10	
Denmark	20	
France	regular consultation fee	440/half day
Georgia	0.5–1.2	
Germany	28 + 8 on Saturday, +35 if performed at home visits, +2–6 issuing a certificate.	120/day
Hungary		210 only on whole weekend days, including fee for nurses
Italy	9–10, 25 at home	31
Netherlands	21, 90 at home, selection work: 2/patients.	
Norway	20	
Poland	15	
Romania	8	18
Serbia		90 once
Slovenia	14	
Switzerland	24–45 (differs between cantons)	
Turkey		200/monthly, for 3 months

**Table 2:** Vaccines used in primary care settings or in vaccination centers.

Country	Manufacturer						
	Astra Zeneca	Jansen	Moderna	Pfizer	Sinofarm	Sputnik	Other
Albania	X			X	X	X	
Austria	X	X	X	X			
Bulgaria	X	X	X	X			
Croatia	X at the beginning only	X	X	X			
Czech R.	X	X	X	X			
Denmark			X	X			
France	X at the beginning	X limited	X	X			
Georgia				X	X		
Germany	X	X	X	X			
Greece	X		X	X			
Hungary	X	X	X	X	X	X only at centers	
Italy			X	X			
Kirgizstan	X	X	X	X	X	X	
Malta	X mainly	X	X	X	X		
Netherlands	X			X			
Norway			X	X			
Poland	X	X	X	X			
Portugalia	X	X	X	X			
Romania	X	X	X	X	X		
Serbia	X		X	X	X		
Slovakia				X			
Slovenia	X	X	X	X			
Sweden	X at the beginning only		X	X			
Swiss			X	X			
Tajikistan	X		X	X		X	
Turkey				X	X		Turkovac
UK	X		X	X			

**Table 3:** Official homepages recommended for GPs at the time of the campaign with information on the respective national vaccination campaign (since then, most of the websites were closed).

Country	Homepage
Albania	shendetesia.gov.al/qendrat-e-vaksinimit-te-hapur
Austria	infogesundheitsministerium.gv.at
	www.salzburg.gv.atwww.impfen
	steiermark.at/cms/ziel/162879324/DE/
	coronainfo.ktn.gv.at/EN/Corona-Impfung-K°%c3%a4rnten
Belgium	www-laatjevaccineren.be
	https://www.sciensano.be/en
Bulgaria	https://www.info-coronavirus.be/nl/coronavirus.bg
Croatia	https://cijepise.zdravlje.hr
France	www.gouvernement.fr/info-coronavirus/carte-et-donnees
Germany	www.moh.gove.ge; https://rki.de
	www.kbv.de/html/covid-19-impfung.php;
	https://www.degam.de/files/Inhalte/Leitlinien
	hausaerzteverbung.de
	kbv.de/html/coronavirus.php
Kirgizstan	www.med.kg; www.vc.emed.gov.kg
Netherlands	www.thuisarts.nl/corona
	www.lhv.nl/actueel/nieuwberichten/coronablog corona.nhg.org/covid19-vaccinatie
	rivm.nl/covid-19-vaccinatie/professionals
	snpg.nl/covid-19-vaccinatie
Norway	www.fhi.no/en/id/vaccines/coronavirus-immunisation20%programme
Portugal	covid19.min-saude.pt-pedido-de-agendamanto
	www.sns.gov.pt-vacinacaocovid19
Romania	www.snmf.ro
	formaremedica.ro
	vaccine-covid.gov.ro
	www.snmf.ro
	formaremedica.ro
	vaccine-covid.gov.ro
Slovakia	www.health.gov.sk/?Ambulantne-ockovanie=ministry20%of20%health-
Slovenia	www.nijz.si/sl/cepljenje-covid
Spain	www.vacunacovid.gob.es
	coronavirus.sergas.gal/contidos/Vacinacion-COVID_profesionais
Sweden	www.1177.se/en/Orebrolan/other-languages/covid-19/
	www.folkhalsomyndigheten.se/the-public-health-agency-of-sweden/
Turkey	hsgm.saglik.gov.tr
	covid19asi.saglik.gov.tr

## Data Availability

The datasets used and analyzed during the current study are available both in printed and electronic form by the corresponding author (I.R.) on reasonable request.

## Appendix A

**Table A1:** Questionnaire.

Your Country	____________________________________
1. Were GPs/FPs in your country involved in the COVID-19 vaccination?	Yes fully/partially No
How were GPs/FPs involved? on voluntary basis, obligation, other, please specify	____________________________________
2. Who were the main providers/executors of vaccination?	Hospitals Health Centers Ad’hoc vaccination points established only for this task Other, please specify ______________
3. On what level was organized the campaign? national, regional, county, city, or walk-in by patients	____________________________________
4. Did the vaccination process require any type of registration from the patients?	Nothing walk-in basis Yes national/regional registration Yes, please specify______________
5. How do you mean, approx. what ratio of vaccines were injected by GPs/FPs?	____________________________________ %
6. Did GPs/FPs receive any reimbursement or fee for injection?	No If yes, how much? ______________ EUR/injection fixed amount, other ______________
7. Which of the vaccines were used by GPs/FPs	ASTRA ZENECA COVID-19 vaccine JANSEN COVID-19 vaccine MODERNA COVID-19mRNA vaccine PFIZER Comirnaty SINOFARM Verocell other______________
8. Could you recommend a national website dealing with the involvement of GPs/FPs?	____________________________________
